# Opposition to Restriction of Immigration

**Published:** 1912-06

**Authors:** 


### Opposition to Restriction of Immigration

WE regret to note that organized opposition is being made to the regulations of the U. S. Government concerning immigration, especially as the opposition is made in the name of foreign nationalities.

The Immigration problem, contrary to what might at first appear, does not involve race problems to any great degree, except in the historic sense of including the forced importation of negroes and in regard to the restriction of Mongolian immigration. The former phase may be regarded as a permanently closed issue. The latter is pretty definitely closed by treaty, although it may prove to be merely latent and perhaps in the sense that latency applies to a volcano between eruptions. At any rate, it is not a phase of the problem with which the present agitation is concerned.

There undoubtedly was a time in the history of the United States when restriction of immigration was a public issue, involving race prejudice, not so much in the sense of a prejudice *against* any particular race as of a prejudice *for* the establishment of a relatively pure race. This time is long past; it is just barely reached by the memories of old men and women; the issue is permanently settled by the fact that about two-thirds of the present population is foreign relatively to the one-third whose fathers, grandfathers and great grandfathers, discussed the pros and cons of "knownothingism" and decided against it, and in favor of what was then denounced as mongrelism.

Almost precisely the same sentiment arose among the Dutch of South Africa and, a decade ago, there was the same struggle between pure and mixed blood and the same ultimate decision though, in the case of the United States adopted by the deliberate vote of the native population, while in the case of the Boers, it was forced by external pressure.

The sentiment in favor of keeping its own blood pure seems to have been instinctive among most primitive peoples although ethnologists are generally sceptic as to the actual purity of the blood of most tribes and nations. The know-nothing sentiment among the Dutch of South Africa was, however, rather more logical than among the Americans of the early nineteenth century. It is true that there had been comparatively little immigration into the northern colonies from about 1700 to about 1830, but the southern colonies as well as Pennsylvania had received considerable accessions only a few years before the Revolution. Most school histories convey the impression that the colonial American is a sort of transplanted Englishman although they state that there were considerable colonies or accessions of Dutch, Swedes, Germans, Scotch, French and others. The colonial American is very far from being a pure blooded Englishman and, if he were, it is worth while considering that the English, at the beginning of the period of American colonization, were a mixture of almost as many races as actually came to the American colonies in the 17th and 18th centuries, that the mixture was not so thorough and that, indeed, the addition of the last important ingredient, the Norman, was not much more remote from the period at which American colonization by the English began, than is that period from the present.

A vastly more important point than the mere history of the way in which the European races have woven their political divisions, is that they were originally a unit and that they have split off into tribes, rejoined one another, again divided and come together in such complicated ways that racial distinctions, as between American, English, French, Dutch, German, etc., amount to about the same thing as streaks in molasses candy while it being being pulled.

What is of infinitely greater importance than race—limiting the discussion to the subdivisions of the Aryans—is the development in an individual or in a family, of certain physical, mental and moral tendencies which differ from the accepted standards of society as a whole. Whether a man is dark or light, is of no consequence as compared to whether he carries with him tubercle bacilli or has trachoma. Whether a woman can pronounce th or z plainly or not, is a very minor point in comparison with her adaptability to decent domestic life on the one hand or prostitution or vagabondage on the other. The flag under which a child has been born in Europe plays no such part as the life to which his forebears have been accustomed for the last five hundred years.

Now it is precisely the physical, mental and moral attributes of the immigrant to which the U. S. Government does pay attention. Once accepted as a citizen or even a permanent resident of the United States, the duty and the self interest of every recent immigrant are precisely the same as for the man who is American for a dozen generations. To demand, as an Italian, or Jew, or Englishman or any other national or racial designation, that undesirable members of the same race be admitted is just as wise as for a newly admitted member of a club or business company to demand that every one else on his street be admitted.

What we want in this country is not the admission nor the exclusion of any particular sub-race, but the admission of the best stock, of any and every nationality, best physically, mentally and morally.

